# Which factors affect the performance of technology business incubators in China? An entrepreneurial ecosystem perspective

**DOI:** 10.1371/journal.pone.0261922

**Published:** 2022-01-11

**Authors:** Xiangfei Yuan, Haijing Hao, Chenghua Guan, Alex Pentland

**Affiliations:** 1 Capital Institute of Science and Technology Development Strategy, Beijing, China; 2 Media Lab, MIT, Cambridge, Massachusetts, United States of America; 3 Computer Information Systems Department, Bentley University, Waltham, Massachusetts, United States of America; 4 School of Economics and Resource Management, Beijing Normal University, Beijing, China; Politecnico di Torino, ITALY

## Abstract

To examine which factors affect the performance of technology business incubators in China, the present study proposes an entrepreneurial ecosystem framework with four key areas, i.e., people, technology, capital, and infrastructure. We then assess this framework using a three-year panel data set of 857 national-level technology business incubators in 33 major cities from 28 provinces in China, from 2015 to 2017. We utilize factor analysis to downsize dozens of characteristics of these technology business incubators into seven factors related to the four proposed areas. Panel regression model results show that four of the seven factors related to three areas of the entrepreneurial ecosystem, namely people, technology, and capital areas, have statistically significant associations with an incubator’s performance when applied to the overall national data set. Further, seven factors related to all four areas have various statistically significant associations with an incubator’s performance in five major regional data set. In particular, a technology related factor has a consistently statistically significant association with the performance of the incubator in both national model and the five regional models, as we expected.

## 1. Introduction

Entrepreneurial ecosystems have become an appealing topic for industrial practitioners, policymakers, and academic scholars to examine entrepreneurship development [1–6]. Many studies have investigated the nature, networks, institutions, and dynamics of entrepreneurial ecosystems to improve the understanding of how to support entrepreneurship growth in different types of economies [7–9]. European scholars studied how entrepreneurial ecosystems are developed and sustained in a holistic way [10–17]. Spigel (2017) believed that the entrepreneurial ecosystem’s boundary should be beyond a firm but within a region [5]. Audretsch and Belitski (2017) investigated variation in entrepreneurial ecosystems in 70 European cities [3]. The present study proposes that a business incubator can be the smallest, atomic entrepreneurial ecosystem because one incubator includes many actors, such as entrepreneurs, mentors, service staff, and investors, who share contextual conditions such as common services, basic infrastructure, local economy, local markets, and regional or national government policies; these comprise an ecosystem with clear boundaries [18–24]. Also, research on the incubator as an entrepreneurial ecosystem can provide more practical and specific policy implications for local governments.

**Fig 1:**
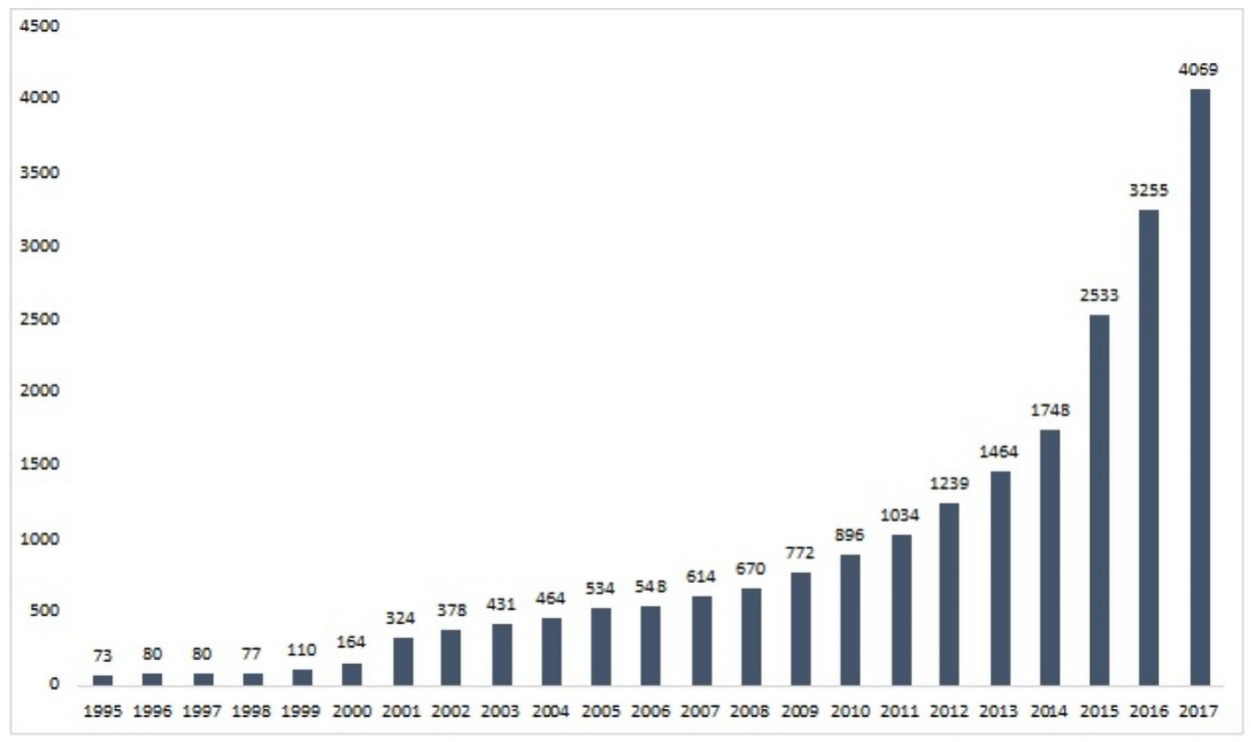
Number of TBIs in China, 1995–2017.

China particularly stresses the importance of the technology business incubator (TBI) in supporting the development of entrepreneurship because of its special history and current economic status, as the second-largest economy and the largest developing economy in the world. Thus, knowing which factors affect the performance of TBIs is particularly critical for China. Chinese governments established and acknowledged hundreds of national level TBIs across China to promote entrepreneurship development [25]. Before the 1980s, China was a purely state-owned central planning economy, and it had no private economy nor entrepreneurship for decades. Even today, the Chinese government and the state owned companies still play important roles in the economy, and annual funding and annual data collections are organized hierarchically from the central government to provincial governments to local governments. Over the past 30 years, technological innovation has been a critical strategy in the Chinese government’s economic reform and development policy, and TBIs are expected to play a primary role in this strategy (Zhang, 2017) [25]. However, the actual development of incubators did not begin until the Mass Entrepreneurship and Innovation Initiative was proposed in 2014, almost 30 years after the Chinese government launched the first TBI, the Wuhan Donghu New Technology Entrepreneurship Center, in 1987 to facilitate knowledge transfer from universities to industry production. Fig 1 shows the number of TBIs in China from 1995 to 2017, which increased sharply between 2014 (1,748 TBIs) and 2017 (4,069 TBIs).

The concept of a business incubator started in the 1950s and has maintained worldwide popularity in entrepreneurship development [26]. The present study concentrates on technology-focused business incubators because the fastest-growing businesses are technology-related. A TBI is a technology-focused business incubator, which aims to support and accelerate the growth and success of startups, and centers on applying novel technologies to industrial applications [27]. Research on TBIs has gained attention worldwide, including the United States [28–32], Europe [33–36], Japan [37, 38], and China [39–42].

Based on the extant research on entrepreneurial ecosystems, the present study proposes four key areas of an entrepreneurial ecosystem that can help evaluate the performance of a TBI; further, we empirically verify those four key areas with a unique panel data set from 857 national level TBIs in China from 2015 to 2017.

The rest of this paper is organized as follows. Section 2 is the theoretical background and hypotheses development. Section 3 describes our data and research methods. Section 4 presents the empirical results. Section 5 discusses the findings and limitations as well as future research opportunities.

## 2. Theory and hypotheses development

### 2.1 Entrepreneurial ecosystems

Entrepreneurial ecosystems have emerged as a theoretical framework that examines and explains the development of regional entrepreneurship and the success of novel startups [5, 7, 18, 21, 22]. The original definition of an ecosystem is a biological community of interacting organisms and their physical environment. Moore (1996) was among the first studies to introduce the concept of ecosystems to business literature and define a business ecosystem as an economic community supported by a group of interacting organizations and individual actors [43].

The World Economic Forum (2013) stated that local and international markets, human capital and financing, mentorship and support systems, robust regulatory frameworks, and major universities are an ecosystem’s most important pillars [11]. Isenberg (2010) identified 13 essential elements of an entrepreneurial ecosystem: leaders, governments, culture, success stories, knowledge, capital, nonprofits and industry associations, educational institutions, infrastructure, geographic locations, networks, venture-oriented professionals, and potential customers [18]. Feld (2012) pointed out nine attributes crucial to a successful entrepreneurial ecosystem: leadership, intermediaries, network density, government, talent, support services, engagement, companies, and capital [44]. Spigel (2017) named 11 important attributes for an entrepreneurial ecosystem: supportive culture, histories of entrepreneurship, work talent, investment capital, networks, mentors and role models, policy and governance, universities, support services, physical infrastructure, and open markets [5]. Chen et al. (2019) examined 85 research articles and extracted 12 widely accepted elements, which either overlapped with or were similar to those of Isenberg (2010) and Spigel (2017) [5, 7, 18]. Audretsch and Belitski (2017) implemented a holistic approach to study the entrepreneurial ecosystem at city level and captured the ecosystem in six domains—culture, formal institutions, infrastructure and amenities, IT, Melting Pot and demand, taking into account regional framework conditions (REDI) and important factors that influence entrepreneurial ecosystem [3].

From the prior studies we find that the essential elements have many overlaps or commonalities, e.g., they all included elements of culture, government, infrastructure, leadership, etc. Further, we find that except for Audretsch and Belitski (2017), who included IT in their framework, Isenberg (2010), Spigel (2017) and other studies missed one important attribute: technology [3, 5, 18]. Audretsch and Belitski (2017) believed that internet technology is critical to business nowadays and they included a technical domain in the entrepreneurial ecosystem framework: internet access and connectivity, which was not included in previous research [3].

We also believe that technology is an important element of an entrepreneurial ecosystem nowadays, and the present study particularly focuses on technology-centered business incubators. Thus, we develop a set of essential elements for an entrepreneurial ecosystem, and categorize them into four key areas: people, technology, capital, and infrastructure; these can be utilized to examine the factors that affect the performance of TBIs.

### 2.2 Four key areas

#### 2.2.1 People area

People area includes the human capital of an entrepreneurial ecosystem, e.g., mentorship, leadership, and supportive services, which are provided by incubators and are essential for incubatee startups to grow. Vanderstraten and Matthyssens (2012) interviewed incubator managers and experts and found that administrative services and personal network by the incubators can strategically position the incubators in the market [45]. Van Weele et al. (2017) found that entrepreneurs may not use an incubator’s resources if they believe that the incubator manager is inexperienced, which means high-quality incubator managers or service people are critical [35]. Thus, we constructed our first hypothesis as follows:

Hypothesis 1: The high-quality/quantity human capital of an entrepreneurial ecosystem will have a positive impact on the performance of a TBI.

#### 2.2.2 Technology area

The technology area represents an incubator’s innovation, which plays a significant role in the establishment and development of technology startups. Technology innovation has been one of the most significant drivers inspiring entrepreneurship development across the world over the last several decades [46, 47]. Audretsch and Belitski (2017) emphasized the importance of information communication technology in supporting entrepreneurial ecosystems as well as in increasing the speed of knowledge spillover [3, 48–50]. Research and development (R&D) investment is a common measure used to evaluate a firm’s innovation [51].

Hypothesis 2: The total R&D investment of an entrepreneurial ecosystem has a positive impact on a TBI’s performance.

In a technology incubator, incubatees are startup firms that focus on how to apply novel technologies, either information technology or biological technology, to production in order to grow the business. A patent is a form of intellectual property that excludes others from making, using, or selling an innovative technology or a unique service in the same field Hence, another common measure used to evaluate or quantify technology innovation is the number of patents or intellectual property applications by a country or an ecosystem (Smith, 2006; Roach and Cohen 2013) [51, 52]. We hypothesize that the number of patents or intellectual property (IP) applications is associated with an entrepreneurial ecosystem’s performance. Accordingly, we formulate the next hypothesis as follows:

Hypothesis 3: The number of patents/patent applications that an entrepreneurial ecosystem owns has a positive impact on a TBI’s performance.

#### 2.2.3 Capital area

The capital area covers various capital or subsidiaries that an entrepreneurial ecosystem receives or owns, which is an essential element driving the success of a startup firm. Venture capital is a type of private equity that investors use to back novel startup companies they believe to have long-term high-growth potential [24]. Thus, we believe capital should be an important area in an entrepreneurial ecosystem.

Previous studies also revealed that government subsidiaries played various roles in support of the development of incubators, e.g., financial support, tax reductions, even direct investment as stakeholders [19, 26, 53–55]; findings from those studies are inconsistent, however. Therefore, we seek to investigate whether various capital supports or subsidies from the government positively impact a TBI’s performance. Thus, we formulate two hypotheses as follows:

Hypothesis 4: Capital support from venture capitalists for an entrepreneurial ecosystem has a positive impact on a TBI’s performance.

Hypothesis 5: Financial support from governments in an entrepreneurial ecosystem has a positive impact on a TBI’s performance.

#### 2.2.4 Infrastructure area

The infrastructure area in the present study refers to the general economic and social infrastructure of an ecosystem at the incubator level, which can include internal infrastructure attributes, such as office space and labs, and external attributes such as regional economy, industrial diversity, and educational institutions near the incubators.

Existing research has found that the local economic environment plays a considerable role in the success of technical business incubators [31, 56, 57]. Regional wealth and the agglomeration of knowledge-intensive business services are positively associated with the level of a region’s economic development [58]. Jacobs’s theory of city development (Jacobs, 1996) assumed that the diversity of industries can promote innovation and regional development and this phenomenon has been demonstrated in many countries [59–63]. In the present study, we use a city’s GDP and industry diversity as an indicator of the economic infrastructure of a TBI [64–66]. We develop the following hypothesis for the relationship between economic infrastructure and an ecosystem’s performance:

Hypothesis 6: A better economic infrastructure in an entrepreneurial ecosystem will have a positive impact on a TBI’s performance.

Educational institutions are regarded as part of social infrastructure [67]. Prior research also revealed that technology startups are more likely to develop a link with educational institutions [68–72], while other research has shown that the interaction between incubated firms and local universities is limited, and not always successful [73–77]. Thus, we developed another hypothesis to examine whether the presence of universities affects the performance of incubators:

Hypothesis 7: Better social infrastructure in an entrepreneurial ecosystem will have a positive impact on a TBI’s performance.

## 3. Data and methods

### 3.1 Data

The data for the present study is a secondary dataset provided by the China Ministry of Science and Technology (MOST), comprising of three waves of surveys of more than 2,000 national-level incubators from about 300 cities across China, spanning 2015 to 2017, with more than 80 variables in each survey. All the CEOs or directors of these TBIs were asked to fill out the survey, which is mostly about the hard facts and objective data about the incubators, as required by MOST. The CEOs double-checked the survey answers filled out by their staff members to ensure data quality and accuracy.

However, every year, some new incubators are established, and some old incubators die out. Considering this, and the various economic developmental levels across China, the present study focuses only on those incubators that have survived all three years of the survey period and are located in provincial capital cities or sub-provincial-level cities, which have relatively broader and stronger industrial bases, better economies, and larger populations. Thus, we narrow down our data set to 857 national-level TBIs in 33 provincial and sub-provincial cities from 28 provinces in China, with the exception of three provinces, Hainan, Qinghai, and Tibet. Further, Liaoning, Shandong, Jiangsu, Guangdong, and Fujian provinceshave two provincial or sub-provincial cities in the data set.

**Fig 2:**
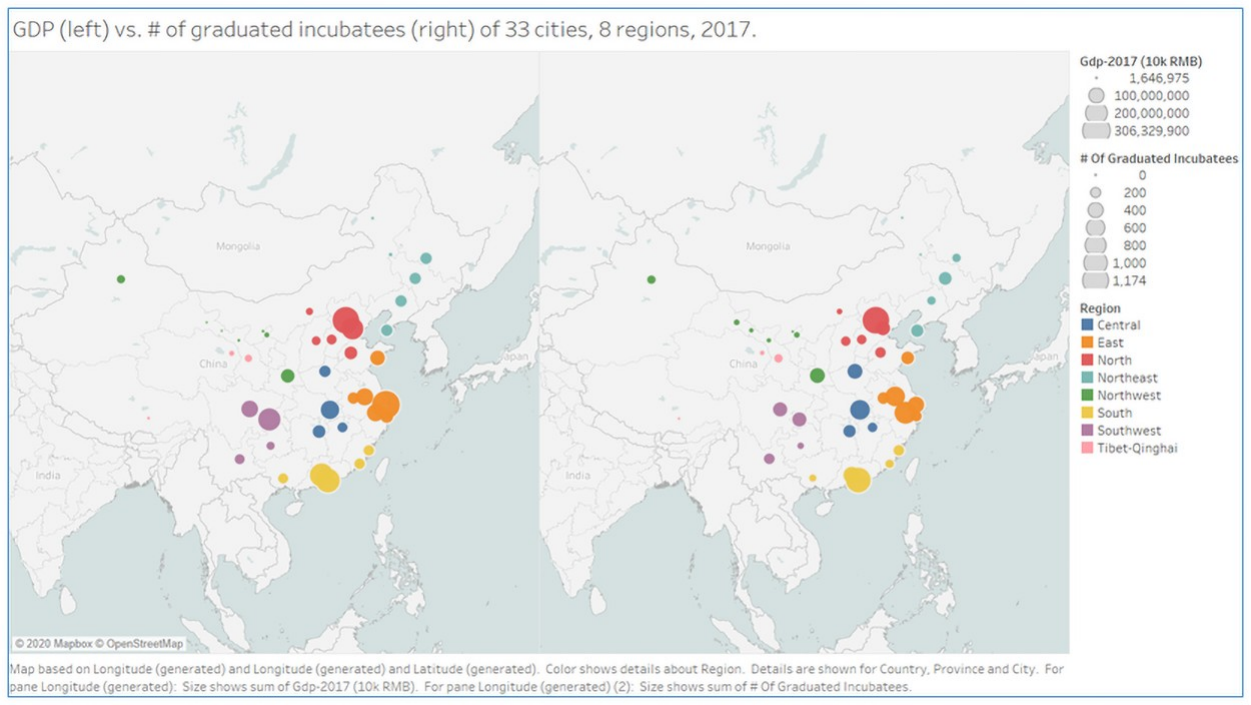
GDP and the number of graduated incubatees in the 33 cities across eight regions, 2017. Created by Tableau.

Entrepreneurial ecosystems are also contingent on regional culture and social-economic backgrounds (Obschonka et al., 2015) [78]. China is one of the largest countries by area in the world, contains a strong regional cultures, and has significantly different levels of economic development across regions; hence, we divide China into eight cultural-geographic regions: Northeast, North China, East China, Central China, South China, Northwest, Southwest, and Gansu, as Fang et al. (2017) suggested [79]. Thus, we can examine the differences among the eight regions in China. In the two Tableau maps that we generated as Fig 2, the circles represent the 33 major cities from our data set, and the eight colors differentiate those cities according to the eight regions as of 2017. The size of the circle represents the GDP amount of each city in the left panel and the number of graduated incubatees in the right panel. In general, we can see three clusters with higher GDP and more graduated incubatees: North China (red circles around the Beijing area), East China (orange circles around the Shanghai area), and South China (yellow circles around the Guangdong area). These two maps provide us with some geographical economic distributions in China.

#### 3.1.1 Dependent variable

How to evaluate the performance of business incubators is a longstanding question in innovation studies, and the literature has used many measures, such as sales revenue, employment growth, or graduation capability [80–83]. The annual number of graduated incubatees from an incubator as the dependent variable is one of the most common measures for an incubator’s performance [31, 38, 39]. The present study adopts this measure to examine the performance of TBIs in China. According to MOST, to graduate from an incubator, the incubatee must satisfy one of the following four criteria:

a) it received the certificate of a “National High-tech Enterprise;” b) it has received accumulated angel investment or venture capital over 5 million RMB yuan; c) its annual revenue exceeded 10 million RMB yuan per year in the last two years; or d) it has been merged, has been acquired or has made an Initial Public Offering (IPO) in either a national or foreign capital market.

#### 3.1.2 Independent variables

Our dataset includes about 88 variables for various data about the incubator, from the physical land area, staff number, and staff education level to the venture capital received, governmental subsidiaries received, R&D investment, IP and patents applied etc., each year. These provide longitudinal comprehensive information on all the national-level incubators in China.

Further, we also combined data from other sources beyond the MOST surveys, e.g., local GDP and local employment data from the China City Statistical Yearbook 2015–2017 and the number of universities in each city from China’s Ministry of Education. However, local GDP is an aggregated value of overall industry, which cannot represent the local industry’s diversity, such as how many sectors there are or the size of each sector. Davies and Tonts (2010) used Shannon’s diversity index (Shannon, 1948) to calculate an industry’s diversity index [84, 85]. We followed Davies and Tonts (2010) and calculated an industry diversity index H for each TBI based on the employment data for the city in which they are located:

$$H=\overset{s}{\underset{i=1}{\sum }}p_{i}\;\text{ln}\;p_{i},$$

where p_i_ is the proportion of people employed in industry section category i and in total for s categories [84].

### 3.2 Methodology

#### 3.2.1 Factor analysis

The MOST dataset contains a large number of variables, making it very challenging to interpret these variables or decide which set of them we should focus on for our analysis. To decrease the large number of variables into a smaller group, and thus reduce the complexity of the data analysis, we apply factor analysis method to acquire a few composite indicators, each of which includes a number of related variables for a certain dimension. Factor analysis has been used by many social science and economic studies to decrease a large number of indicators into a smaller number of factors for a more straightforward economic interpretation [3, 86]. The basic idea behind factor analysis is that the variability of many observable variables may reflect a smaller number of unobserved factors.

#### 3.2.2 Evaluating a TBI’s performance: Panel regression

Given our unique panel data, we can run two types of panel data regression model, i.e., the fixed effects model or the random effects model. The fixed effects model allows us to control for time-invariant variables that we cannot observe or measure, such the unmeasurable quality of each specific business incubator or unavailable variables relating to an incubator’s idiosyncratic heterogeneity. If the variation across entities is random and uncorrelated with the independent variables, then we should use the random-effects regression model. Either a fixed effects model or a random effects model has its own empirical interpretation. In practice, a Hausman test is conducted to distinguish between the random effects model and fixed effects model. If the Hausman test is statistically significant, the fixed effects model is preferred to the random effects regression model.

## 4. Results

### 4.1 Descriptive statistics

In Table 1, we present the descriptive statistics of a number of important variables for the national data set and the eight regional data sets. Table 1 shows that the average number of graduated incubatees of each incubator in each year is 6.22 at the national level, and the national median is 4. Of the eight regions, North, Central, and Southwest are higher than the national average, while Northeast, East, South, Northwest, and Qinghai-Tibet are lower. At the national level, the maximum number of IP applications in a year is 1,320, and this is the highest number of IP applications an incubator could make in a year, from an incubator in the East Region, the most developed region in China. The national average of the number of IP applications is 64, and the national median is 30. For the number of IP applications, North, Central, South, Northwest, and Southwest regions are all higher than the national average. Further, the descriptive statistics vary significantly across regions.

**Table 1:** Descriptive statistics of key variables of our dataset.

	Variables	Number of IP applications by incubatees	Number patents by incubatees	Number of incubator’s full time staff	Number of incubator’s full time staff who have higher education	Industries diversity index of the city	Number of colleges and universities in the city	Number of graduate firms listed on the stock market	Number of incubatees received venture capital	Number of graduate incubatees every year (dependent variable)
All Regions	# of Observations	2571	2571	2571	2571	2571	2571	2571	2571	2571
	Mean	63.88	105.86	17.68	16.67	2.39	34.65	1.45	17.56	6.22
	Median	30	46	14	13	2.41	31	0	6	4
	Std. Dev	99.81	168.47	14.53	13.58	0.18	17.83	3.45	36.34	8.05
	Min	0	0	0	0	1.77	6	0	0	0
	Max	1320	2171	204	198	2.71	72	36	542	81
Northeast	# of Observations	255	255	255	255	255	255	255	255	255
	Mean	32.62	64.51	18.04	16.85	2.50	25.96	0.40	7.84	4.73
	Median	9	17	13	12	2.57	27	0	2	3
	Std. Dev	84.6	144.11	16.04	15.11	0.13	2.96	1.33	13.18	7.10
	Min	0	0	0	0	2.28	20	0	0	0
	Max	940	1338	94	94	2.65	29	12	61	68
North	# of Observations	390	390	390	390	390	390	390	390	390
	Mean	74.9	130.87	20.81	19.38	2.55	46.98	2.06	23.25	8.16
	Median	40.5	64	16	15	2.7	67	0	10	5
	Std. Dev	101.4	178.89	14.83	13.73	0.18	20.84	3.88	42.61	9.35
	Min	0	0	2	1	2.19	10	0	0	0
	Max	835	1270	120	120	2.71	67	31	453	55
East	# of Observations	978	978	978	978	978	978	978	978	978
	Mean	57.38	90.38	15.51	14.84	2.34	31.97	0.95	15.98	5.01
	Median	27	40	12	12	2.35	35	0	6	2
	Std. Dev	91.24	143.8	12.28	11.52	0.1	8.35	2.15	26.2	7.08
	Min	0	0	0	0	2.00	8	0	0	0
	Max	1320	1873	204	198	2.43	38	23	201	81
Central	# of Observations	213	213	213	213	213	213	213	213	213
	Mean	91.65	135.87	21.23	20.16	2.32	32.92	3.11	27.15	9.60
	Median	56	77	17	15	2.31	25	1	15	7
	Std. Dev	116.98	162.65	14.38	14.27	0.10	10.61	4.98	39.61	9.69
	Min	0	0	4	4	2.04	23	0	0	0
	Max	802	1105	83	81	2.50	46	35	243	61
South	# of Observations	417	417	417	417	417	417	417	417	417
	Mean	65.66	116.8	16.00	14.68	2.27	42.42	1.55	12.85	5.53
	Median	25	42	12	11	2.46	72	0	2	7
	Std. Dev	112.07	205.65	17.97	16.12	0.27	30.4	4.09	33.66	8.35
	Min	0	0	0	0	1.77	7	0	0	0
	Max	1258	2171	202	180	2.53	72	30	305	61
Northwest	# of Observations	96	96	96	96	96	96	96	96	96
	Mean	91.4	173.14	22.18	21.34	2.51	32.63	2.76	33.45	5.69
	Median	59	84	20	18	2.52	42	1	13	4
	Std. Dev	110.23	247.29	17.84	17.66	0.05	15.15	6.2	75.56	5.89
	Min	0	0	0	5	2.45	6	0	0	0
	Max	480	1306	120	120	2.63	42	36	430	32
Southwest	# of Observations	177	177	177	177	177	177	177	177	177
	Mean	77.33	113.68	19.14	18.29	2.42	24.10	1.62	21.02	8.89
	Median	44	59	17	26	2.46	25	0	6	6
	Std. Dev	95.72	150.61	10.47	9.8	0.09	3.15	3.47	54.32	9.40
	Min	0	0	2	2	2.22	18	0	0	0
	Max	571	776	59	55	2.55	27	24	542	51
Qinghai-Tibet	# of Observations	45	45	45	45	45	45	45	45	45
	Mean	27.07	42.49	18.69	17.4	2.50	17	0.96	8.42	5
	Median	7	9	20	18	2.5	17	0	4	3
	Std. Dev	45.2	71.83	7.21	7.32	0.01	0	2.65	14.32	7.54
	Min	0	0	2	2	2.49	17	0	0	0
	Max	175	298	34	30	2.50	17	11	60	39

The total number of observations for all three years in the Northwest, Southwest, and Qinghai-Tibet is 96 (32 incubators), 177 (69 incubators), and 45 (15 incubators), respectively, which are rather small. Thus, we will drop those three regions from our regression analysis because of their limited sample size, which may bias the regression model results.

### 4.2 Main factors

Table 2 presents the results of our factor analysis. The Kaiser-Meyer-Olkin (KMO) test was performed to evaluate the fitness of our factor analysis, and the test result is 0.775, which means our factor analysis suits this data set well (Kaiser, 1974) [87]. Our factor analysis identified seven factors that collectively explained 82.7% of the total variance. Table 2 shows the loadings from our factor analysis, which are the correlation coefficients between the variables (rows) and factors (columns).

**Table 2:** Result of factor analysis.

Factors	People-service	People-mentor	Technology-capital	Capital	Infrastructure-capital	Infrastructure-GDP	Infrastructure-diversity-education
Incubator’s investment on shared technology platform	0.15	-0.02	0.02	0.07	0.11	0.01	-0.04
Incubatees’ investment on R&D	0.13	0.05	**0.52**	0.10	0.15	0.08	-0.01
Number of IP applications by incubatees	0.15	0.03	**0.85**	0.01	-0.001	-0.01	-0.03
Number of patents by incubatees	0.16	0.05	**0.86**	0.04	0.05	0.002	-0.03
Number of purchased abroad patents	0.03	0.02	0.19	0.09	0.02	0.06	-0.04
Number of national level R&D projects	0.01	-0.03	0.03	0.02	0.04	-0.04	0.001
Number of incubator’s full time staff	**0.98**	0.01	0.11	0.03	0.03	0.01	0.01
Number of incubator’s full time staff who havehigher education	**0.98**	0.04	0.14	0.03	0.01	-0.02	0.001
Number of incubator’s staff who received skill training	**0.36**	0.16	0.26	-0.01	-0.04	-0.06	0.01
Average graduate periods of incubatees	0.10	0.08	0.29	0.12	0.02	-0.08	-0.02
Number of incubatees	0.13	0.22	0.31	0.11	0.21	-0.03	0.02
Incubated fund from incubator	0.16	0.10	0.16	0.24	0.05	0.04	0.04
External venture capital received by the incubatees	0.11	0.06	**0.38**	0.14	-0.07	0.06	0.13
Incubating area	0.15	0.03	0.34	0.12	**0.44**	-0.07	-0.06
Incubators’direct investment from government	0.10	0.07	0.24	0.11	**0.35**	-0.06	-0.06
Incubators’ subsidies from government	0.21	0.05	0.20	0.14	0.14	0.10	-0.02
Incubators’ tax reduction from government	0.14	0.17	0.15	0.05	0.001	-0.0001	-0.06
Incubatees’ subsidies from government	0.08	0.08	0.34	0.20	0.13	0.06	-0.09
Number of entrepreneurship advisors	0.13	**0.51**	0.21	0.08	-0.01	-0.02	0.01
Number of contracted professional services	0.13	**0.41**	0.29	0.11	0.13	-0.04	0.03
Number of training sessions for incubatees	0.10	0.24	0.10	0.12	-0.02	0.01	-0.001
Number of graduate firms listed on the stock mareket	0.25	0.06	**0.50**	**0.45**	0.15	0.01	0.04
Number of incubatees which received venture capital	0.15	0.23	**0.52**	**0.42**	0.04	0.01	0.02
GDP per capita of the city’s urban area	-0.02	-0.01	0.01	0.01	-0.03	**0.65**	-0.15
Industry diversity index of the city	0.04	0.03	-0.06	-0.01	0.003	-0.34	**0.76**
Number of colleges and universities of the city	-0.02	-0.002	0.003	0.02	-0.03	0.20	**0.74**

Note: The number of observations = 2571, pooled data for 857 incubators over three years.

The first factor loadings are quite heavy on variables such as the number of staff members of an incubator (0.98), the number of staff members who completed higher education (0.98), and the number of incubator staff members who received work skill training (0.36). Those variables are obviously associated with the quantity and quality of a TBI’s people area, and more about the service staff, and we label this factor as the “people-service factor.”

The second factor shows a strong connection with the number of entrepreneurship advisors (0.51) and the number of contracted professional services (0.41) in an incubator. Thus we call the second factor the “people-mentor factor.”

The third factor loads heavily on a few variables, such as research and development (R&D) investment (0.52), the number of intellectual property applications (0.85), and the number of patents held by all incubatees of an incubator (0.86), which are related to the technology area. Further, this factor demonstrates a strong relationship with the number of incubatees that received venture capital (0.52) and the number of incubatees listed on the stock market (0.50), which is consistent with our expectation that an incubatee firm with a higher potency in the technology area, i.e., a high number of IP applications or patents or high investment in R&D, is associated with achieving better outcomes, i.e., receiving more venture capital or being publicly listed. In addition, this factor is also related to the external venture capital received by the incubatees (0.38), which can be categorized as the capital area. Thus, we name this factor the “technology-capital factor” because it is associated with the technology and capital areas.

The fourth factor is heavily loading on the number of graduate firms listed (0.45) on the Chinese stock exchange market and the number of incubatees that received venture capital (0.42), and the venture capital overlaps with the third factor. It is also associated with another variable, i.e., funds from the incubator itself (0.24). Thus, we categorize this factor into the capital area, and we call it the “capital factor.”

The fifth, the sixth, and the seventh factors are all related to the infrastructure area. The fifth factor loading is more associated with the incubating area (0.44), which is related to infrastructure area because it is the physical infrastructure of an incubator, and incubators’ direct investment from government (0.35), which belongs to the capital area. Thus, the fifth factor is a mixture of the infrastructure area and the capital area, and we call it the “infrastructure-capital factor.”

The sixth factor loading is heavily on GDP per capita (0.65) of the city where the TBIs locate, which is related to a city’s local economic infrastructure. Thus, we categorize this factor as the infrastructure area, and call it the “infrastructure-GDP factor.”

The seventh factor exhibits a strong association with the industry diversity index (0.76) and the number of universities in the city (0.74) where the TBI locates. The industry diversity index of a region is related to the local economic infrastructure because, usually, the more diversified a region’s industry, the more mature its economic infrastructure (Quigley,1998) [65]. In general, social infrastructure includes hospitals, schools, and universities, which contribute to a region’s public life; the number of colleges and universities indicates the strength of a region’s social infrastructure in research (Pradhan & Rawlings, 2002; Latham & Layton, 2019) [67, 88]. Hence, we categorize this factor as the infrastructure area and call it the “infrastructure factor.”

### 4.3 Regression results

Based on the factor analysis results from the previous section, we run fixed effects and random effects panel regression models with the seven factors as follows:

$$loggrad_{it}=\alpha +\beta_{1}People\_service_{it}+\beta_{2}People\_mentor_{it}+\beta_{3}Tech\_capital_{it}+\beta_{4}Capital_{it}+\beta_{5}Infra\_capital_{it}+\beta_{6}Infra\_GDP_{it}+\beta_{7}Infrastructure_{it}+u_{it},$$ (1)

where loggrad_it_ is the log of the number of graduated incubatees in incubator i in year t (we take the log in order to adjust the abnormal distribution of variable values), People_service_it_ represents the factor associated with the people-service area of incubator i in year t, People_mentor_it_ represents the factor associated with the people-mentor area of incubator i in year t, Tech_capital_it_ represents the factor associated with the technology-capital area of incubator i in year t, Capital_it_ represents the factor associated with the three capital areas of incubator i at year t, Infra_capital_it_ represents the factor associated with the mixture of infrastructure and capital area of incubator i at year t, Infra_GDP_it_ represents the factor associated with GDP-related infrastructure area of incubator i at year t, Infrastructure_it_ represents the factor associated with the infrastructure area of incubator i in year t, and the last term, u_it_, serves as the error term.

Because the descriptive statistics in Table 1 show that some incubators had zero graduated incubatees in certain years, we take the log of the number of graduated incubatees in Eq (2) to adjust the zeros for the logarithm values:

$$\text{log}grad_{it}=\text{log}(grad_{it}+1)$$ (2)

As previously discussed, the above panel regression model (1) is run with six different data sets, i.e., one data set for the entire country’s TBI data and five data sets for five regions. The six model results are presented in Table 3. Based on Hausman tests, we can see that the national model, Northeast, East, and South models are the fixed effects models, which means the incubators of those models had strong and constant fixed effects over the three-year time period. The North and Central regions’ models are random effects models, which means the incubators’ effects are uncorrelated to individual incubator characteristics. The R-squared for all models is satisfactory.

**Table 3:** Results of panel regression models.

	People-service	People-mentor	Technology-capital	Capital	Infrastructure-capital	Infrastructure-GDP	Infrastructure-diversity-education	Constant	Hausman Test	FE/RE	Over-all R^2^	# of ob. /incubators	# of cities
**All Regions**	0.28** (0.11)	0.40** (0.14)	0.62** (0.12)	0.74** (0.17)	0.13 (0.17)	-0.15 (0.12)	-0.23 (0.39)	0.08* (0.04)	0.005	Fixed effects	0.17	2571/857	33
**Northeast**	1.53** (0.48)	0.88 (0.71)	2.31* (0.94)	3.82** (1.23)	-0.24 (0.72)	-2.71* (1.15)	0.27 (1.5)	-0.86 (0.95)	0.030	Fixed effects	0.09	255/85	4
**North**	0.09 (0.14)	0.57** (0.18)	0.69** (0.13)	0.35* (0.19)	0.2 (0.2)	-0.42* (0.24)	0.05 (0.16)	0.74** (0.19)	0.480	Random effects	0.16	390/130	6
**East**	0.33* (0.16)	0.45 (0.3)	0.97** (0.26)	0.75* (0.38)	0.97** (0.39)	0.16 (0.37)	-0.56 (0.94)	-0.47** (0.2)	0.050	Fixed effects	0.26	978/326	6
**Central**	0.37** (0.14)	0.49* (0.24)	0.55** (0.14)	0.17 (0.18)	-0.02 (0.21)	-0.22 (0.41)	0.48 (0.38)	1.37** (0.18)	0.659	Random effects	0.18	213/71	4
**South**	0.67 (0.4)	0.48 (0.4)	1.07** (0.31)	1.06* (0.44)	0.31 (0.7)	-0.16 (0.16)	-3.79** (1.38)	-0.54** (0.2)	0.015	Fixed effects	0.14	417/139	5

Notes:

* indicates p < 0.05 and

** indicates p < 0.01.

## 5. Discussions

### 5.1 National model

**Fig 3:**
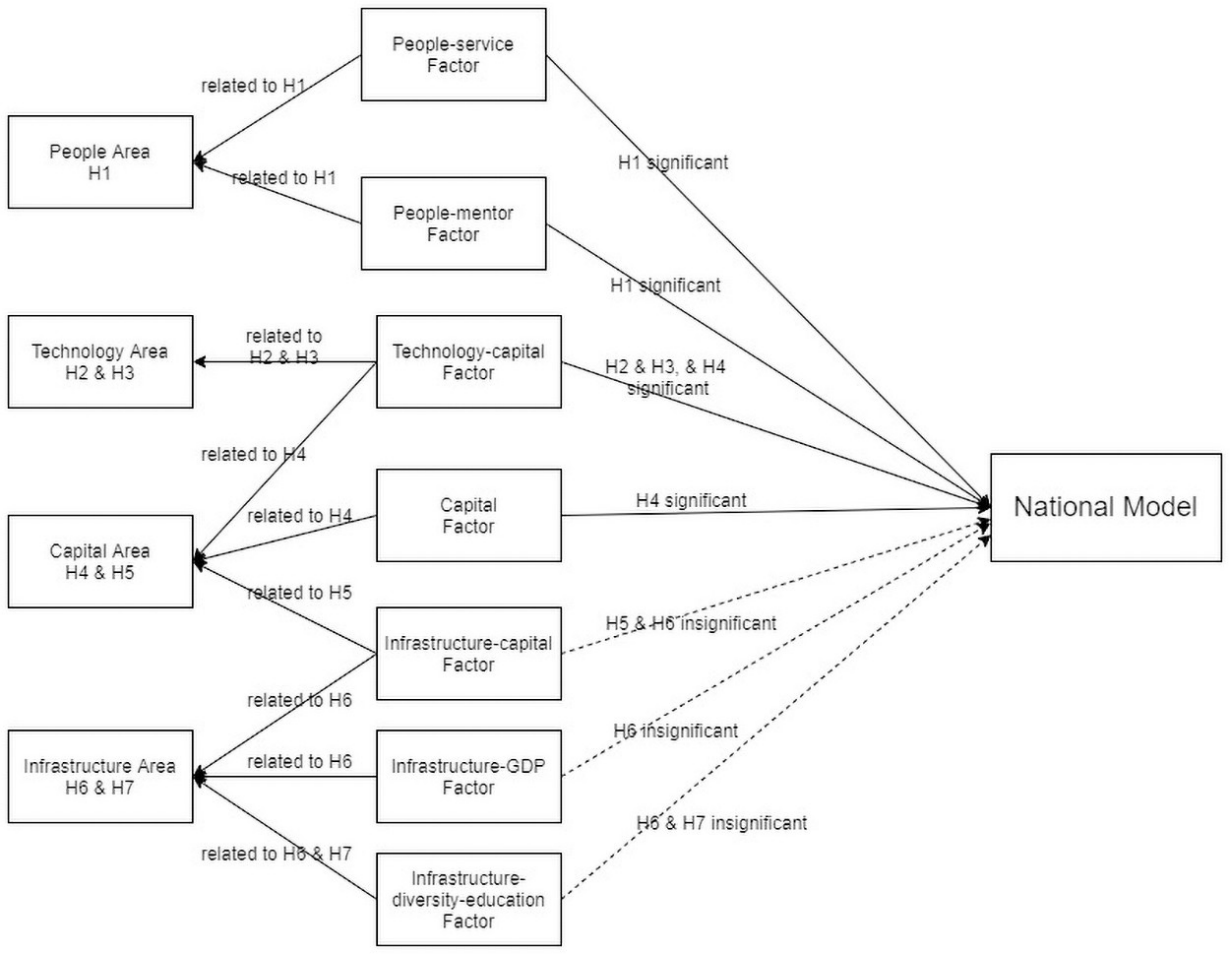
National regression model results and the hypotheses.

Based on the panel regression result in Table 3, Fig 3 summarizes the relationships among the seven factors and the four key areas, along with the significance of the seven factors on the seven hypotheses about the overall national data set. The model result shows that four of seven factors, i.e., the people-service factor, people-mentor factor, technology-capital factor, and capital factor, related to three areas, i.e., people area, technology area, and capital area, have statistically significant positive effects on the TBI’s performance, after controlling for the TBI’s fixed effects. This means that, when a TBI has a high score for any of the above four factors, the TBI will have a high number of graduated incubatees. These findings match the authors’ expectations based on our practical experiences with incubators in China, as well as extant research from China and other countries.

If a TBI has a high people-service factor score, which indicates it has high-quantity or high-quality staff, or if a TBI has a high people-mentor factor score, which means it has high number of entrepreneurship advisors and many contracted professional services, then the TBI will have more successfully graduated incubatees. This result verifies our Hypothesis 1: High-quality/quantity human capital will improve the performance of an entrepreneurial ecosystem.

If a TBI has a high technology-capital factor score, which means it has more IP applications/patents and high investment in R&D, then the incubator will have more successful graduated incubatees. This finding also resonates with our expectations and verifies Hypotheses 2 and 3, about the technology area: The total R&D investment of an ecosystem and the number of patents/patent applications within an ecosystem both have a positive impact on the ecosystem’s performance. Based on our knowledge, the relationship between intellectual property/patents and the performance of an entrepreneurial ecosystem has not been studied in prior research, probably because of data limitations (Fagerberg & Srholec, 2008) [86].

If a TBI has a high capital factor score, which means the TBI will have a high number of graduate firms listed on the stock market and a high number of incubatees that received venture capital from investors, then the TBI will have more graduated incubatees. The positive effects of this capital factor are related to our Hypotheses 4 and 5, about the capital area: Capital support from venture capitalists and financial support from governments both have a positive impact on an ecosystem’s performance.

However, none of the three infrastructure related factors, i.e., infrastructure-capital factor, infrastructure-GDP factor, and infrastructure factor, are statistically significant in the national model, which may suggest that the basic infrastructure of a TBI, such as the incubator area size, local GDP per capita, local industry diversity, or higher education environment, does not have a statistically significant impacts on the performance of a TBI. This may indicate that most technical incubatees nowadays do not need a large office or factory area to build a successful business in information technology or biological science because of their special knowledge intensive characteristics in this modern age. Further, the finding that a government’s investment or subsidies do not play a role in the success of TBIs, is consistent with Yin (2009), which revealed that a beneficial government policy was not effective for TBI development in China [89].

Also, the infrastructure-GDP area, which scores highly on GDP per capita in a city’s urban area, has a strong correlation with regional economic development but may not be tied to a city’s innovation driver. One explanation for this phenomenon, according to Marshall (1890), is that regions with a specialized industry, unlike those with diversified industry, tend to be more innovative because knowledge spillover is easier between similar nearby companies [90]. Further, over the last few decades, TBIs have focused on technologies such as the Internet, high tech, biomedicine, etc. Thus, unrelated industry diversity or general traditional industry diversity does not benefit the innovation of TBIs [59]. Another explanation may be that China’s regional variation is too strong, which effectively dilutes industry diversity in this aggregated national data set. In the following section, we will investigate and discuss the regional model results.

Regarding the relationship between the number of universities and innovation within a region, prior research has found that the geographic connection between educational institutions and innovation may no longer hold due to the increased mobility of educated people within a country (Florida, 2002; Florida et al., 2008) [91, 92]. Similarly, Xiao and North (2018) found that the number of universities in a city does not have a significant impact on the scale or the type of innovation activity in incubators in China [39].

### 5.2 Regional model

**Fig 4:**
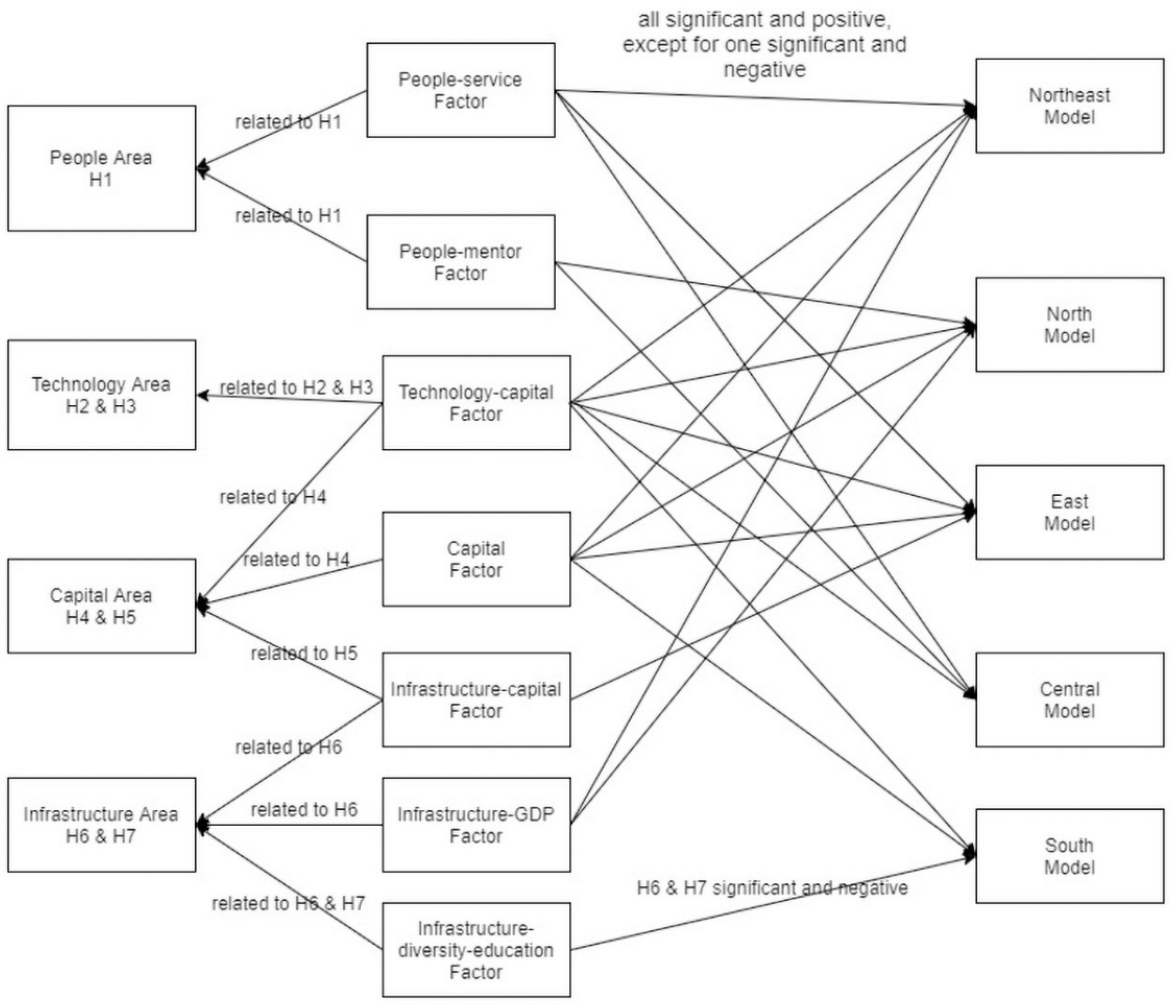
Five regional regression model results and the hypotheses.

Based on the regression model results presented by Table 3, Fig 4 summarizes the relationships among the seven factors and the four key areas, along with the significance of the seven factors on the seven hypotheses based on regression model results for the five regional data sets.

The people-service factor has a statistically significant effect in Northeast, East, and Central China, but not in North and South China. These results verify Hypothesis 1. This statistical significance is also consistent with our national model results from the previous section. The people-mentor factor shows a statistically significant impact on the TBI’s performance in two regions, i.e., North and Central China, which are less developed regions compared with the South, Northeast, and East China. This result is also consistent with our national model, and it supports Hypothesis 1. Regarding the reasons for the regional differences, one explanation might be that China has significant differences in regional economy and culture; however, this assumption will need further study, with more data or longer time periods.

The technology-capital factor is statistically significant and positive in all five regional models; it is the only factor that has a statistically significant positive association with the TBI’s performance in both the five regional models and the national model. This matches our expectations because we think technology should play a very important role in a technology-centered business incubator, or a technology-centered entrepreneurial ecosystem. The technology-capital factor is related to the technology area and the capital area, and is the only factor related to the technology area. It is exciting to see this factor consistently significant across all regional models because technology and capital are the keys to success for any TBI. The significant association between the technology-capital factor and the success of a TBI is a new finding, to the best of the authors’ knowledge, and it has not been discussed in extant studies. Indeed, this finding makes sense because the major goal of a TBI is to incubate high-tech startup firms and to help those nascent technical companies gain maturity or graduate In sum, the higher the technology-capital factor score of a TBI is, the more IP applications and patents are owned by the incubatees of a TBI, the more R&D investment received by incubatees, the more graduated firms are listed on the stock market, the more venture capital received by incubatees, then the more graduated incubatees of the TBI. The technology-capital factor’s significant positive effects on TBI’s performance supports our Hypotheses 2, 3, and 4.

Another capital area related factor is the capital factor, which exhibits positively statistically significant influence on the performance of a TBI four of the five regions, Northeast, North, East, and South China as well as in the national model. This means, in an incubator, that the more graduated incubatees are listed on the stock market and the more incubatees received venture capital, the better performance of a TBI. This finding supports our Hypotheses 4 and 5.

All three factors related to infrastructure area are not statistically significant in the national model, but some are statistically significant in some regional models. The infrastructure-capital factor shows a statistically significant impact on the performance of a TBI in the East region, which supports our Hypotheses 5 and 6. The infrastructure-GDP factor has a statistically significant impact on a TBI’s performance in the Northeast and North regions, which supports our Hypothesis 6. The infrastructure-diversity-education factor has no statistically significant impact on a TBI’s performance any region, except for South China, where it has a negative effect. This interesting negative impact may suggest that cities with more universities or more diversified industry are less innovative. This finding relates to our Hypotheses 6 and 7. The question of why there is such a negative effect needs more data and research, in a separate study.

## 6. Contributions and limitations

### 6.1 Contributions

The present study makes a few contributions. First, we develop a new entrepreneurial ecosystem framework to examine the performance of a TBI, focusing on four key areas: people, technology, capital, and infrastructure areas. Previous studies have focused on various forms of the essential elements or areas of an entrepreneurial ecosystem, such as people, capital, or infrastructure [7, 11, 18, 44], but besides Audretsch and Belitski (2017), who emphasized the importance of internet technology for entrepreneurial ecosystems, most research on entrepreneurial ecosystems has ignored the technology area [3]. We particularly believe that technology should be an important element in examining entrepreneurial ecosystems. Our empirical results also showed that the technology area related factor is the only factor with a statistically significant impact on TBI’s performance in both the national model and the five regional models.

Second, empirically, we find encouraging and exciting results by utilizing a three-year longitudinal data set from Ministry of Science and Technology, China, which verifies that our proposed framework can be used to examine the relationship between the four key areas of an entrepreneurial ecosystem and the success of a TBI. Our regression model results show that regional difference is significant, probably because of China’s large geographic area, long and uneven economic development history, and diversified local culture.

Finally, our research also expands empirical study on innovations in China, which is the second-largest economy and the largest transforming developing economy in the world. Based on our factor analysis, government support does not appear to be a statistically significant factor among over eighty variables. This may suggest that, following about 40 years of economic reform, the market economy, rather than the state-owned economy, may become the primary driver in the development of innovation in China. This finding is consistent with the findings of Yin (2009) [89].

### 6.2 Limitations and future research

The present study has limitations. First, it is based on empirical data from TBIs in large cities in China, as there are not sufficient data from smaller cities; thus, interpretation of the model results should be careful and limited. The proposed framework can be extended to examine a TBI’s performance in smaller cities if more data from such cities becomes available. Second, another direction for future research would be to conduct onsite interviews to further investigate the why some factors have significant relationship with a TBI’s performance, and more detailed qualitative interpretations are needed. Third, the present study’s results are based on empirical data from China. Future research can expand our theoretical framework to other countries’ to conduct a cross-country comparison study.

## References

[1] Autio E., Nambisan S., Thomas L. D., and Wright M. Digital affordances, spatial affordances, and the genesis of entrepreneurial ecosystems. Strategic Entrepreneurship Journal. 2018; 12(1), 72–95.

[2] Audretsch DB, Belitski M, Cherkas N. Entrepreneurial ecosystems in cities: The role of institutions. PLoS ONE. 2021; 16(3): e0247609. doi: 10.1371/journal.pone.0247609 33684163 PMC7939368

[3] Audretsch DB, and Belitski M. Entrepreneurial ecosystems in cities: establishing the framework conditions. Journal of Technology Transfor. 2017; Vol. 42, 1030–1051.

[4] Chandra Y. Mapping the evolution of entrepreneurship as a field of research (1990±2013): A scientometric analysis. PLoS ONE. 2018; 13(1): e0190228. doi: 10.1371/journal.pone.0190228 29300735 PMC5754054

[5] Spigel B. The Relational Organization of Entrepreneurial Ecosystems. Entrepreneurship Theory and Practice. 2017; Vol.41: 1, page(s): 49–72.

[6] Roundy PT, Brockman B, and Bradshaw M. The resilience of entrepreneurial ecosystems, Journal of Business Venturing Insights. 2017; 8: 99–104.

[7] Chen J, Cai L, Bruton DG and Sheng N. Entrepreneurial ecosystems: what we know and where we move as we build an understanding of China, Entrepreneurship & Regional Development. 2019.

[8] Roundy PT, Bradshaw M., and Brockman BK. The emergence of entrepreneurial ecosystems: A complex adaptive systems approach. Journal of Business Research. 2018; 86, 1–10.

[9] Mack E. and Mayer H. The evolutionary dynamics of entrepreneurial ecosystems. Urban studies. 2016; 53(10), 2118–2133.

[10] Stam E, and Nooteboom B. Entrepreneurship, innovation and institutions. In Audretsch D., Falck O., & Heblich S. (Eds.), Handbook of research on innovation and entrepreneurship. Cheltenham: Edward Elgar. 2011; 421–438.

[11] World Economic Forum. Entrepreneurial ecosystems around the globe and company growth dynamics. Davos: World Economic Forum. 2013.

[12] Acs ZJ, Autio E, and Szerb L. National systems of entrepreneurship: Measurement issues and policy implications. Research Policy. 2014; 43(3), 449–476.

[13] Autio E, Kenney M, Mustar P, Siegel D, and Wright M. Entrepreneurial innovation: The importance of context. Research Policy. 2014; 43(7), 1097–1108.

[14] Levie JD., and Autio E. Hard facts or soft insights? Fact-based and participative approaches to entrepreneurship ecosystems policy analysis and management. Paper prepared for the workshop organised by the Henley Centre for Entrepreneurship, December 2014.

[15] Ghio N, Guerini M, Lehmann EE, and Rossi-Lamastra C. The emergence of the knowledge spillover theory of entrepreneurship. Small Business Economics. 2014; doi: 10.1007/s11187-014-9588-y

[16] Audretsch DB, and Lehmann EE. The seven secrets of Germany. Oxford: Oxford University Press. 2016.

[17] Theodoraki C, Karim M, and Audretsch DB. The Effectiveness of Incubators’ Co-Opetition Strategy in the Entrepreneurial Ecosystem: Empirical Evidence From France. IEEE TRANSACTIONS ON ENGINEERING MANAGEMENT. Nov. 2020.

[18] Isenberg D. The Big Idea: How to Start an Entrepreneurial Revolution, Harvard Business Review, 2010/06/01.

[19] Amezcua AS, Grimes MG, Bradley SW, Wiklund J. Organizational sponsorship and founding environments: a contingency view on the survival of business-incubated firms, 1994–2007. Academy of Management Journal. 2013; 56(6),1628–1654.

[20] Motoyama Y and Knowlton K. “Examining the Connections within the Startup Ecosystem: A Case Study of St. Louis,” Entrepreneurship Research Journal, De Gruyter, 2017; vol. 7(1), pages 1–32, January.

[21] Theodoraki C., and Messeghem K. Exploring the entrepreneurial ecosystem in the field of entrepreneurial support: a multi-level approach. International Journal of Entrepreneurship and Small Business. 2017; 31(1), 47–66.

[22] Theodoraki C, Messeghem K, and Rice MP. A social capital approach to the development of sustainable entrepreneurial ecosystems: an explorative study. Small Business Economy. 2018; 51, 153–170.

[23] Dagnino. GB. The academic incubator as a fluid Mosaic: an ecological interpretive framework, in Nicoló, D. (Ed.): Start-Ups and Start-Up Ecosystems: Theories, Models and Case Studies in the Mediterranean Area. 2015; pp.79–89, ASERS Publishing.

[24] Malecki EJ. Entrepreneurship and Entrepreneurial ecosystems, Investopedia, 2020. https://www.investopedia.com/terms/v/venturecapital.asp.

[25] Zhang Z. China Business Incubation 30 Years:1987-2017, Beijing: Science and Technology Documentation Press. 2017.

[26] Mian S, Lamine W, and Fayolle A. Technology Business Incubation: An overview of the state of knowledge. Technovation. 2016; 50-51, 1–12.

[27] Hansen EG, Grosse-Dunker F. and Reichwald R. Sustainability Innovation Cube: A Framework to Evaluate Sustainability-Oriented Innovations, International Journal of Innovation Management. 2009; 13(4), pp. 683–713.

[28] Mian SA. Assessing value-added contributions of university technology business incubators to tenant firms. Research Policy. 1996;25(1996),325–335.

[29] Mian SA. Assessing and managing the university technology business incubator: An integrative framework. Journal of Business Venturing. 1997; 12(4),251–285.

[30] Rice MP. Co-production of business assistance in business incubators: an exploratory study. Journal of Business Venturing. 2002; 17(2), 163–187.

[31] Harper-Anderson E and Lewis DA. What Makes Business Incubation Work? Measuring the Influence of Incubator Quality and Regional Capacity on Incubator Outcomes. Economic Development Quarterly. 2018; 32(1), 60–77.

[32] Olkiewicz M, Wolniak R, Eva-Grebski M, and Olkiewicz A. Comparative Analysis of the Impact of the Business Incubator Center on the Economic Sustainable Development of Regions in USA and Poland. Sustainability. 2019;11(173); doi: 10.3390/su11010173

[33] Bergek Aand Norrman C. Incubator best practice: a framework. Technovation. 2008; 28, 20–28.

[34] Albort-Morant G. and Oghazi P. How useful are incubators for new entrepreneurs? Journal of Business Research. 2016; 69, 2125–2129.

[35] van Weele M, van Rijnsoever FJ, and Nauta F. You can’t always get what you want: How entrepreneur’s perceived resource needs affect the incubator’s assertiveness. Technovation. 2017; 59, 18–33.

[36] Lukeša M, Longo MC, and Zouhar J. Do business incubators really enhance entrepreneurial growth? Evidence from a large sample of innovative Italian start-ups. Technovation. 2018; doi: 10.1016/j.technovation.2018.07.008

[37] Tsai HY, Chung TT, and Liu RR. A field study on business incubator in Japan: a new type of “co-working space”. Journal of Organizational Innovation (Online). 2017; 10(1),173–184.

[38] Fukugawa N. Is the impact of incubator’s ability on incubation performance contingent on technologies and life cycle stages of startups? evidence from Japan. International Entrepreneurship and Management Journal. 2018; 14, 457–478.

[39] Xiao L and North D. The graduation performance of technology business incubators in China’s three tier cities: the role of incubator funding, technical support, and entrepreneurial mentoring. Journal of Technology Transfer. 2017; 42(2017):615–634.

[40] Xiao L and North D. The role of Technological Business Incubators in supporting business innovation in China: a case of regional adaptability? Entrepreneurship and Regional Development. 2018; 30:1–2, 29–57, doi: 10.1080/08985626.2017.1364789

[41] Li Q and Liu X. Incubator Innovation Efficiency Evaluation and Relationship Research. Science and Technology Management Research (China). 2018; 4, 59–63.

[42] Hong J, Chen M, Zhu Y, and Song G. Technology business incubators and regional economic convergence in China. Technology Analysis and Strategic Management. 2016; 29(6):1–14.

[43] Moore JF. The Death of Competition: Leadership and Strategy in the Age of Business Ecosystems. Harper Business. 1996.

[44] Feld B. Startup Communities: Building an Entrepreneurial Ecosystem in Your City. Wiley ISBN: 978-1-118-48331-2. 2012.

[45] Vanderstraeten J and Matthyssens P. Service-based differentiation strategies for business incubators: Exploring external and internal alignment, Technovation. 2012; Volume 32, Issue 12, 2012, Pages 656–670.

[46] Branstetter L. Intellectual Property Rights, Innovation, and Development: Is Asia Different?” Branstetter L., Millennial Asia. 2017; 8 (4), April 2017, pp. 5–25.

[47] Branstetter L, Drev M, and Kwon N. Get with the Program: Software-Driven Innovation in Traditional Manufacturing. Management Science. 2018; 7 Feb 2018.

[48] Zacharakis A, Shepard D, and Coombs J. The development of venture-capita-backed internet companies: An ecosystem perspective. Journal of Business Venturing. 2003; 18, 217–231.

[49] Li K, Godley A, Belitski M, Li W, and Manwani S. The importance of e-leadership in meeting digital challenges. 2015. Computerweekly.com. March 1, 2016.

[50] Acs ZJ, Braunerhjelm P, Audretsch DB, and Carlsson B. The knowledge spillover theory of entrepreneurship. Small Business Economics. 2009; 32(1), 15–30.

[51] Smith K. Measuring Innovation, The Oxford Handbook of Innovation Edited by Jan Fagerberg and David C. Mowery. 2006. Print Publication Date: Jan 2006.

[52] Roach M and Cohen WM. Lens or Prism Patent Citations as a Measure of Knowledge Flows from Public Research. Management Science. 2013; 59 (2) 504–525. doi: 10.1287/mnsc.1120.1644 24470690 PMC3901515

[53] Chandra A, He W, and Fealey T. Business Incubators in China: A Financial Services Perspective. Asia Pacific Business Review. 2007; 13. 79–94.

[54] Pergelova A and Angulo-Ruiz F. “The impact of government financial support on the performance of new firms: the role of competitive advantage as an intermediate outcome.” Entrepreneurship and Regional Development. 2014; 26.9-10: 663–705.

[55] Guan C and Yuan X. Direct investment, Financial Subsidies and Tax Incentives——Comparative Analysis Based on National Incubator Data. Beijing Social Science. 2018; 8,107–119.

[56] Moya-Clemente I, Ribes-Giner G, and Pantoja-Díaz O. Identifying environmental and economic development factors in sustainable entrepreneurship over time by partial least squares(PLS). PLoS ONE 2020;15(9): e0238462. doi: 10.1371/journal.pone.0238462 32886680 PMC7473577

[57] Pe’er A and Keil T. Are all startups affected similarly by clusters? Agglomeration, competition, firm heterogeneity, and survival. Journal of Business Venturing. 2013; 28(3), 354–372.

[58] Pan F, Zhao SXBV, and Dariusz W. The rise of venture capital centres in China: A spatial and network analysis. Geoforum. 2016; 75:148–158.

[59] Boschma R, and Iammarino S. Related Variety, Trade Linkages, and Regional Growth in Italy. Economic Geography. 2010; 85(3):289–311.

[60] Wang J. Agglomeration economy, correlation variety and urban economic growth: Empirical analysis based on panel data of 279 cities at prefecture level or above. Journal of Finance and Economics. 2016; 5,135–144.

[61] Fritsch M and Kublina S. Related variety, unrelated variety, and regional growth: The role of absorptive capacity and entrepreneurship. Regional Studies. 2018; 52(10), 1360–1371.

[62] Haqa I. and Zhu S. Does export variety determine economic growth in Pakistan? Applied Economics Letters. 2018.

[63] Content J, Frenken K, and Jordan J. Empirical analysis of the effects of related variety at national and regional level in EU, EU Working Paper. 2018.

[64] Barro RJ and Sala-i-Martin X. Economic Growth. McGraw-Hill, New York. 1995.

[65] Quigley JM. Urban diversity and economic growth. Journal of Economic Perspectives. 1998; 12(2), 127–138.

[66] Chong SK, Bahrami M, Chen H, Balcisoy S, and Bozkaya B. Economic outcomes predicted by diversity in cities. EPJ Data Science. 2020; 9(1), 17.

[67] Pradhan M and Rawlings LB. The impact and targeting of social infrastructure investments: Lessons from the Nicaraguan Social Fund. The World Bank Economic Review. 2002; 16(2), 275–295.

[68] Audretsch DB and Belitski M. Science Parks and Business Incubation in the United Kingdom: Evidence from University Spin-Offs and Staff Start-Ups. In: Amoroso S., Link A., Wright M. (eds) Science and Technology Parks and Regional Economic Development. Palgrave Advances in the Economics of Innovation and Technology. Palgrave Macmillan, Cham. 2019.

[69] Roach M. Encouraging entrepreneurship in university labs: Research activities, research outputs, and early doctorate careers. PLoS ONE. 2017; 12(2): e0170444. doi: 10.1371/journal.pone.0170444 28178270 PMC5298308

[70] Colombo MG and Delmastro M. How effective are technology incubators? Evidence from Italy. Research Policy. 2002;31 (2002),1103–1122.

[71] Phillimore J. Beyond the linear view of innovation in science park evaluation An analysis of Western Australian Technology Park. Technovation. 1999; 19.11 (1999): 673–680.

[72] Gozali L, Masrom M, Zagloel TYM, Haron HN, Garza-Reyes JA, Tjahjono B, et al. Performance Factors for Successful Business Incubators in Indonesian Public Universities. International Journal of Technology. 2020; 11(1), pp. 155–166.

[73] Quintas P, Wield D, and Massey D. Academic-industry links and innovation: questioning the science park model.” Technovation. 1992; 12.3: 161–175.

[74] Vedovello C. Science parks and university-industry interaction: Geographical proximity between the agents as a driving force. Technovation. 1997; Volume 17, Issue 9, Pages 491–531.

[75] Bakouros YL, Mardas D, and Varsakelis N. Science park, a high tech fantasy?: an analysis of the science parks of Greece. Technovation. 2002; 22.2 (2002): 123–128.

[76] Westhead P and Storey DJ. Links between Higher Education Institutions and High Technology Firms. Omega. 1995; 23, 345–360. doi: 10.1016/0305-0483(95)00021-F

[77] Rubin TH, Tor Helge A, and Stead A. Knowledge flow in Technological Business Incubators: Evidence from Australia and Israel. Technovation. 2015; 41–42: 11–24.

[78] Obschonka M, Stuetzer M, Gosling SD, Rentfrow PJ, Lamb ME, Potter J, et al. Entrepreneurial Regions: Do Macro-Psychological Cultural Characteristics of Regions Help Solve the “Knowledge Paradox” of Economics? PLoS ONE. 2015. 10(6): e0129332. doi: 10.1371/journal.pone.0129332 26098674 PMC4476658

[79] Fang C, Liu H, and Luo K. Comprehensive regionalization of human geography in China. Acta Geographica Sinica. 2017; 72(2), 179–196.

[80] Hackett SM and Dilts DM. A Systematic Review of Business Incubation Research. Journal of Technology Transfer. 2004; 29(1), 55–82.

[81] Albort-Morant G. and Ribeiro-Soriano D. (2015) A Bibliometric Analysis of International Impact of Business Incubators. Journal of Business Research, 69, 1775–1779. doi: 10.1016/j.jbusres.2015.10.054

[82] Voisey P, Gornall L, Jones P, Thomas B. The measurement of success in a business incubation project. Journal of Small Business and Enterprise Development. 2006; Vol 13, No 3, pp 454–468.

[83] Torun M, Peconick L, Sobreiro V, Kimura H, and Pique J. Assessing business incubation: A review on benchmarking, International Journal of Innovation Studies. 2018;Volume 2, Issue 3, Pages 91–100.

[84] Davies Aand Tonts M. Economic Diversity and Regional Socioeconomic Performance: An Empirical Analysis of the Western Australian Grain Belt. Geographical Research. 2010; 48(3), 223–234.

[85] Shannon CE. A Mathematical Theory of Communication. The Bell System Technical Journal. 1948; 27,379–423, 623–656.

[86] Fagerberg J and Srholec M. National innovation systems, capabilities and economic development, Research Policy. 2008; 37 (2008), 1417–1435.

[87] Kaiser HF. An index of factorial simplicity. Psychometrika. 1974; 39(1), 31–36.

[88] Latham A and Layton J. Social infrastructure and the public life of cities: Studying urban sociality and public spaces. 2019;First published: 20 June 2019, 10.1111/gec3.12444.

[89] Yin Q. On Factors in the Development of Incubators of Scientific and Technological Enterprises—Based on the Data Analysis of Enterprises in Incubation in Jiangsu Province. Jiangsu Social Science. 2009; 4228-231.

[90] Marshall A. Principles of economics (8th Ed.). London: Macmillan. 1890.

[91] Florida Richard. (2005). Cities and the creative class, Psychology Press.

[92] Florida R, Mellander C, and Stolarick K. Inside the black box of regional development—human capital, the creative class and tolerance. Journal of Economic Geography. 2008; 8(5), 615–649.

